# More Is Not Always Better: Interventions for Caregivers of Older and Dependent Relatives

**DOI:** 10.3390/jcm11113010

**Published:** 2022-05-26

**Authors:** Javier López

**Affiliations:** Department of Psychology, School of Medicine, Universidad San Pablo-CEU, CEU Universities, 28925 Alcorcón, Madrid, Spain; jlopezm@ceu.es; Tel.: +34-913724700

## 1. Introduction

The population all around the world is becoming older. Population aging is one of the greatest challenges of the 21st century. An increasing number of older adults are reaching an age at which physical and cognitive decline begins and family caregivers become their primary providers of care.

Psychological treatment for caregivers is one of the most important factors related to gerontology. Mental practitioners should demonstrate competence in treating caregivers’ burden, anxiety, and depression.

Despite the increased number of caregivers, which implies a growing need for psychological interventions, a small proportion of psychologists decide to specialize in working with them. Understanding time use (more or less) in psychological treatments is important for designing treatments that optimize their implementation among caregivers.

## 2. Discussion

It seems that family members who care for an elder adult relative at home are a difficult access group. Many are not interested in participating in the different interventions because they are too burdened, and others, even if they are interested, have serious difficulties that prevent them from becoming involved. Moreover, usually, caregivers show little initiative to use formal resources [1,2].

Therefore, it seems essential to adapt the interventions to caregivers’ specific needs and characteristics (especially their limited availability of time and their overload of chores). In this sense, data indicate that those long interventions and/or having numerous measurements generate a higher number of dropouts. For this reason, offering short intervention programs which do not represent a factor of added stress is needed. The duration of the intervention programs varies greatly. However, in most cases, the sessions are spread over approximately 8 h [1].

Noriega et al. [3] developed a caregivers´ treatment during a weekend with sessions of spa therapy (4.5 h) with an optional addition of psychoeducation (7.5 h). This is obviously less than the two-week duration recommended by Karagülle and Karagülle [4]. Most caregivers’ interventions are time-limited, and some researchers have successfully developed minimal therapist contact treatments [5,6].

Reduced treatments have some risks, but they also offer many potential advantages over standard treatments: First, a reduced treatment eliminates the need for prolonged and frequent intervention visits, improving access to treatment for many caregivers. Specifically for rural caregivers who must travel significant distances for attending the treatment sessions, a decrease in clinic visits makes obtaining treatment more feasible due to a reduction in travel costs and lost work time. A reduction in visits, however, will likely improve access to care for all caregivers, not just those living in rural areas. For example, people in big cities such as Madrid or Barcelona spent around 50–60 min in public transportation daily. Moreover, some caregivers may be unable to attend frequent intervention visits due to fluctuations in the health of their dependent old relatives. Furthermore, after the initial reduced treatments, more intervention visits may be conducted if the interventionist believes more progress can be made toward goal stress levels.

In any case, considering the chronically stressful nature of the caregiving scenario, it is not surprising that the stress and coping model of Lazarus and Folkman [7] has been the conceptual framework within which several studies aimed at understanding psychological distress in this context. This model highlights not only the stressful context and the emotional consequences for the caregivers but also the relevance of personal resources for understanding the differences in distress between individuals (e.g., anger and spiritual meaning, Márquez-González et al. [8]; motives for caring, Romero-Moreno et al. [9]; optimism, López et al. [10]; self-efficacy, Peñacoba et al. [11]; resilience and emotional intelligence, Gómez-Trinidad et al. [12]; and spirituality, López, Romero-Moreno et al. [13]). The effects achieved by the different interventions in the improvement in emotional distress are, when they occur, moderate. However, since caring is a chronic stressor, whose presence is maintained during and after the intervention, it is not surprising that it is difficult to modify the emotional distress. In these circumstances, it can be considered an achievement to ensure that emotional distress does not increase.

As previous caregiving research points out, we can conclude that interventions focused on caregivers are beneficial, although they do have limited utility [1]. Therefore, it seems necessary to develop programs adapted to caregivers´ needs, focused on some specific personal resources (e.g., anger and spiritual meaning, motives for caring, optimism, self-efficacy), in which effective strategies are provided for managing the stress that the care situation entails. In this way, caregivers´ well-being may improve and, therefore, that of the older adults they care for. In addition, the better state of caregivers can in turn delay the institutionalization of the elderly, thus contributing to this “aging at home” that, beyond constituting a political objective, is a vital desire for many people.
